# A systematic review of mitochondrial abnormalities in myalgic encephalomyelitis/chronic fatigue syndrome/systemic exertion intolerance disease

**DOI:** 10.1186/s12967-020-02452-3

**Published:** 2020-07-29

**Authors:** Sean Holden, Rebekah Maksoud, Natalie Eaton-Fitch, Hélène Cabanas, Donald Staines, Sonya Marshall-Gradisnik

**Affiliations:** 1 National Centre for Neuroimmunology and Emerging Diseases (NCNED), Menzies Health Institute Queensland, Griffith University, Gold Coast, Australia; 2 School of Medicine, Griffith University, Gold Coast, Australia; 3 Consortium Health International for Myalgic Encephalomyelitis, Griffith University, Gold Coast, Australia; 4 School of Medical Science, Griffith University, Gold Coast, Australia

**Keywords:** Myalgic Encephalomyelitis, Chronic Fatigue Syndrome, Systemic Exertion Intolerance Disease, Mitochondria, Energy metabolism

## Abstract

**Background:**

Patients with Myalgic Encephalomyelitis/Chronic Fatigue Syndrome (ME/CFS) or Systemic Exertion Intolerance Disease (SEID) present with a constellation of symptoms including debilitating fatigue that is unrelieved by rest. The pathomechanisms underlying this illness are not fully understood and the search for a biomarker continues, mitochondrial aberrations have been suggested as a possible candidate. The aim of this systematic review is to collate and appraise current literature on mitochondrial changes in ME/CFS/SEID patients compared to healthy controls.

**Methods:**

Embase, PubMed, Scopus and Medline (EBSCO host) were systematically searched for articles assessing mitochondrial changes in ME/CFS/SEID patients compared to healthy controls published between January 1995 and February 2020. The list of articles was further refined using specific inclusion and exclusion criteria. Quality and bias were measured using the Joanna Briggs Institute Critical Appraisal Checklist for Case Control Studies.

**Results:**

Nineteen studies were included in this review. The included studies investigated mitochondrial structural and functional differences in ME/CFS/SEID patients compared with healthy controls. Outcomes addressed by the papers include changes in mitochondrial structure, deoxyribonucleic acid/ribonucleic acid, respiratory function, metabolites, and coenzymes.

**Conclusion:**

Based on the included articles in the review it is difficult to establish the role of mitochondria in the pathomechanisms of ME/CFS/SEID due to inconsistencies across the studies. Future well-designed studies using the same ME/CFS/SEID diagnostic criteria and analysis methods are required to determine possible mitochondrial involvement in the pathomechanisms of ME/CFS/SEID.

## Background

Myalgic Encephalomyelitis/Chronic Fatigue Syndrome (ME/CFS), more recently termed Systemic Exertion Intolerance Disease (SEID) is a complex multidimensional illness where patients present with a variety of pathophysiological symptoms including immunological, endocrine and neurological disruption [1–4]. Symptom presentation is heterogeneous ranging from mild to severe, even leaving some patients bed bound [1]. The underlying pathomechanisms of ME/CFS/SEID are nebulous and the search for standardised biomarkers continues, so diagnosis entirely depends upon symptom specific case criteria following the exclusion of any other explanatory diagnosis [1–4].

There are four main criteria used to diagnose ME/CFS/SEID: the 1994 Fukuda Criteria (FC), 2003 Canadian Consensus Criteria (CCC), 2011 International Consensus Criteria (ICC), and 2015 Institute of Medicine Criteria (IOMC). The FC, CCC, ICC and IOMC all specify fatigue as the cardinal symptom [1–4]. As fatigue is a key diagnostic symptom for ME/CFS/SEID, energy metabolism may be a significant pathomechanistic factor. Mitochondrial function is an important aspect of energy metabolism and has been the focus of recent study [5–23].

Mitochondria are maternally inherited multifunctional organelles that play a critical role in energy harvesting, transformation and storage as well as other intracellular signaling processes [24]. Residing within the inner mitochondrial membrane is the electron transport chain (ETC). The ETC consists of five multi-subunit enzyme complexes (complexes I through V) and two electron carriers: coenzyme Q10 (CoQ_10_) and cytochrome c which are involved in oxidative phosphorylation and subsequent production of adenosine triphosphate (ATP) [24]. Mitochondria are also fundamental for immune processes such as inflammasome activation and general intracellular calcium signaling [25, 26]. Due to their physiological importance, mitochondria are implicated in a wide variety of pathological conditions including ME/CFS/SEID [5–23].

The aim of this systematic review is to present and appraise current research that has compared ME/CFS/SEID patient participants to healthy control (HC) participants and the role mitochondria may have in ME/CFS/SEID pathology. Foci include variations in mitochondrial deoxyribonucleic acid (mtDNA), messenger ribonucleic acid (mRNA), mitochondrial respiratory function, metabolites, and coenzymes. [5–23]. Literature on this topic will help guide prospective studies in the search for an appropriate biomarker for this debilitating illness.

## Methods

### Literature search

**Fig. 1:**
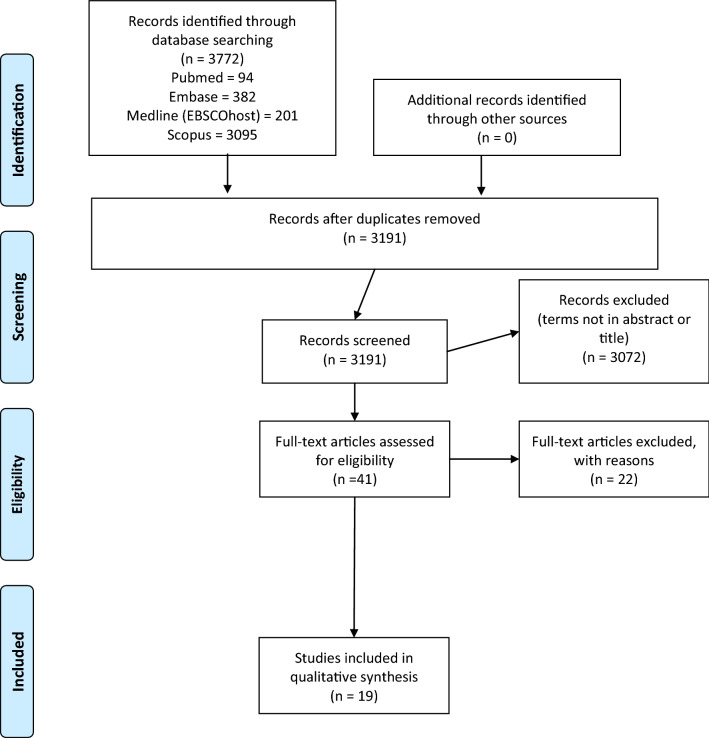
PRISMA flow diagram of literature search for included studies in this review of mitochondria and ME/CFS/SEID

This systematic review was conducted according to the Preferred Reporting Items for Systematic Reviews and Meta-Analyses (PRISMA) (Fig. 1) and Cochrane guidelines. PRISMA and Cochrane guidelines were used to ensure international standards were maintained and used for reporting information contained in this systematic review. The databases EMBASE, PubMed, Scopus and Medline (EBSCO host) were systematically searched using full-text and Medical Subject Headings (MeSH) terms. Mitochondrial search terms and ME/CFS/SEID search terms are presented in Table 1. Boolean operators ‘OR’ and ‘AND’ were used to expand the search to include all relevant key terms and to specify articles containing both a ME/CFS/SEID search term and a  mitochondrial search term. Full code can be found in Additional File 1. Literature searches were conducted independently by authors SH and RM on February 18th, 2020. Reference list checking and citation searching was carried out, no additional papers were found. Unpublished literature was not searched. No additional papers were identified in the final search or through alternative databases such as Griffith University institute library or Google Scholar.

**Table 1:** Title and abstract screening terms

Mitochondrial search terms (14 terms)	ME/CFS/SEID search terms (23 terms)
Mitochondria	Chronic fatigue syndrome
Mitochondrion	Myalgic encephalomyelitis
Mitochondrial contraction	Encephalomyelitis, myalgic
Contraction, mitochondrial	Chronic fatigue syndromes
Contractions, mitochondrial	Fatigue syndromes, chronic
Mitochondrial contractions	Chronic fatigue-fibromyalgia syndrome
Mitochondri*	Chronic fatigue fibromyalgia syndrome
Energy metabolism	Chronic fatigue-fibromyalgia syndromes
Pyruvate dehydrogenase	Fatigue-fibromyalgia syndrome, chronic
Electron transport chain	Fatigue-fibromyalgia syndromes, chronic
ATP	Postviral fatigue syndrome
ADP	Infectious mononucleosis-like syndrome, chronic
TCA cycle	Infectious mononucleosis like syndrome, chronic
Citric acid cycle	Royal free disease
	Chronic fatigue and immune dysfunction syndrome
	Chronic fatigue disorder
	Chronic fatigue disorders
	Fatigue disorder, chronic
	Fatigue disorders, chronic
	Systemic exertion intolerance
	Fatigue syndrome, postviral
	Fatigue syndromes, postviral
	Postviral fatigue syndromes

### Inclusion and exclusion criteria

Studies were included for review if at least one mitochondrial search term AND at least one ME/CFS/SEID search term (Table 1) were found in the title or abstract and the study complied with the following inclusion criteria: (i) published after 1994 (ii) research conducted on human participants only, aged 18 years or older; (iii) full- text article was available in the English language; (iv) original research only was reported; (v) ME/CFS/SEID was diagnosed using: FC (1994), CCC (2003), ICC (2011) or IOMC (2015); (vi) investigation was conducted on mitochondrial aberrations in ME/CFS/SEID patients compared to a HC group.

Articles that did not contain at least one mitochondrial search term AND at least one ME/CFS/SEID search term in the title or abstract were excluded from the review (Table 1). Articles were excluded if any of the following applied: (i) written prior to the introduction of the FC on December 15th 1994 (all studies 1994 or earlier were excluded considering time was required to be aware of the FC); (ii) conducted in non-human participants or those under the age of 18; (iii) articles not written in English or not available as full-text; (iv) studies that reported on non-original data including: duplicate studies, case reports or review articles; (v) use of criteria other than FC, CCC, ICC or IOMC; (vi) Comparison with a patient group (e.g.,) fibromyalgia or depression, without comparison to HC participants; (vii) studies that were not within the scope of this review.

### Selection of studies

Following retrieval of articles from the databases all articles were stored in the reference management software package Endnote X9.2. Duplicates were manually removed and all articles that did not contain the listed key words in title or abstract were omitted. The remaining articles were reviewed and those that followed the eligibility criteria were selected. This process was conducted by authors SH and RM independently. Final papers to be included in this review were then reassessed by all other listed authors.

During title and abstract screening, we performed manual and automated screening. The automated screening was achieved by building an algorithm in Endnote 9.2 using the grouping function with AND/OR logic gates. When we compared the manual screening result to automated screening, one article was retained by automated screening which manual screening had excluded, that article was included in final analysis. Two articles were found by manual screening which automated screening had excluded, both of which were included in final analysis. Authors SH and RM then checked discrepancies, the final list of articles retained after screening was deemed accordant and correct by both authors.

### Data extraction

Following selection of papers, data was extracted including: (i) study design; (ii) diagnostic criteria used; (iii) sample size; (iv) method of analysis.

### Quality assessment

The Joanna Briggs Institute (JBI) Critical Appraisal Checklist for Case Control Studies (CACCCS) checklist was used to evaluate quality and bias (Additional File 2). CACCCS checklist was selected based on it being an internationally recognized, validated, rigorous process to evaluate study quality and bias. Quality assessment was conducted by authors SH and RM independently. Each checklist item assesses the following: [1] group matching, [2] source population, [3] criteria, [4] method of exposure, [5] assessment of exposure, [6] confounding variables identification, [7] mitigation of confounding variables, [8] measurement of outcomes, [9] exposure period selection, [10] statistical analysis. Items four, five and nine of the JBI CACCCS were not assessed as intervention-based studies were excluded from analysis.

## Results

A total of 3772 papers were identified from Medline (EBSCOhost) (201), Embase (382), PubMed (94) and Scopus (3095). All duplicates were removed, and the remaining papers were screened according to inclusion and exclusion criteria. Following this process, the total number of articles were refined to 19 [5–23]. The selection process as conducted by PRISMA guidelines has been summarized in Fig. 1.

### Overview of papers

The study characteristics of the 19 papers included in this review are summarized in Table 2. All papers in this review were observational case control studies that examined mitochondria in ME/CFS/SEID patients compared with HC participants [5–23]. No potentially relevant papers were excluded from this review.

**Table 2:** Summary of study characteristics

Author	Year	Study design	Dx	Sample sizes		Method of analysis
				ME/CFS/SEID	HCs	
Armstrong et al.	2015	Observational case–control	Canadian criteria	34	25	NMR spectroscopy
Billing-Ross et al.	2016	Observational case–control	Fukuda criteria	193	196	Illumina sequencing
Booth et al.	2012	Observational case–control	Fukuda criteria	138	53	ATP profile test
Castro-Marrero et al.	2013	Observational case–control	Fukuda criteria	23	15	Western blot bioluminescence assay
Germain et al.	2017	Observational case–control	Fukuda criteria IOM 2015	17	15	Mass spectrometry
Light et al.	2013	Observational case–control	Fukuda criteria	39	22	Real time QPCR
Maes et al.	2009	Observational case–control	Fukuda criteria	58	22	High Performance Liquid Chromatography
Mandarano et al.	2019	Observational case–control	Canadian criteria	53	45	Seahorse XFe96, Flow cytometry, Confocal microscopy
Missailidis et al.	2020A	Observational case–control	Canadian criteria	51	22	MitoTracker Green FM, Seahorse XFe24 (mitochondrial stress test)
Missailidis et al.	2020B	Observational case–control	Canadian criteria	51	22	Seahorse XFe24 (mitochondrial stress test), XF Glycolysis stress test
Naviaux et al.	2016	Observational case–control	Canadian criteria Fukuda criteria IOM 2015	45	39	Hydrophilic interaction liquid chromatography, electrospray ionization, and tandem mass spectrometry
Nguyen et al.	2016	Observational case–control	Fukuda criteria	17	19	Flow cytometry
Nguyen et al.	2019	Observational case–control	Fukuda criteria International Consensus criteria	6	6	Seahorse XFp
Plioplys and Plioplys	1995	Observational case–control	Fukuda criteria	15	15	Electron microscopy
Shungu et al.	2012	Observational case–control	Fukuda criteria	15	13	Magnetic resonance spectroscopy
Sweetman et al.	2019	Observational case–control	Canadian criteria	10	10	RNA sequencing
Tomas et al.	2017	Observational case–control	Fukuda criteria	52	35	Seahorse XFp
Venter et al.	2019	Observational case–control	Fukuda criteria	UK: 89 moderate, 29 severe RSA: 143 moderate	UK: 64 RSA:98	DNA sequencing
Yamano et al.	2016	Observational case–control	Fukuda criteria	Training: 47 Validation: 20	Training: 46 Validation: 20	Agilent CE capillary electrophoresis system

*ATP* adenosine triphosphate, *DNA* deoxyribonucleic acid, *HCs* healthy controls, *IOM* Institute of Medicine, *ME/CFS/SEID* myalgic encephalomyelitis/chronic fatigue syndrome/systemic exertion intolerance disease, *NMR* nuclear magnetic resonance, *QPCR* quantitative polymerase chain reaction, *RSA* Republic of South Africa; *RNA* ribonucleic acid, *UK* United Kingdom

### Participant and study characteristics

Participant characteristics are summarized in Table 3. The average number of ME/CFS/SEID patients across all papers was 57.8 and the average number of HC participants was 40.75. Most participants were female (77%). Six of the studies reported race, wherein the largest proportion of participants were Caucasian [6, 8, 12–14, 17]. The average ages across all studies were 43.7 for ME/CFS/SEID patients and 42.4 for HC participants. Six of the studies included ME/CFS/SEID patients that met the CCC [5, 12, 13, 18, 22, 23], 13 studies used the FC as a minimum requirement for inclusion [6–9, 11, 13–17, 19–21], one study used FC and IOMC [9] and another study used the FC and ICC [15]. The remaining study used a combination of the FC, CCC and IOMC [13]. The average illness duration for ME/CFS/SEID patients was 15.1 years.

**Table 3:** Summary of participant characteristics

Reference	Dx	Sample (n)		Age (years, average (SD)		Sex, female (%)		Illness duration	Marker assessed	Sample source
		ME/CFS/SEID	HC	ME/CFS/SEID	HC	ME/CFS/SEID	HC			
Armstrong et al. [5]	Canadian Criteria	34	25	34.9 (1.8 SE)	33.0 (1.6 SE)	100%	100%	NR	Metabolites	Whole blood and urine
Billing-Ross et al. [6]	Fukuda Criteria, Canadian Criteria	193	196	NR	NR	NR	NR	NR	mtDNA	DNA
Booth et al. [7]	Fukuda Criteria	138	53	Cohort 1: 45.1 (11.8) Cohort 2: 41.1 (12.1)	35.9 (13.4)	Cohort 1: 79% Cohort 2: 70%	76%	NR	ATP	Neutrophils
Castro-Marrero et al. [8]	Fukuda Criteria	23	15	44.1 (3.8)	43.5 (5.4)	65.2%	66.7%	15.6 (10.8)	CoQ10, ATP, lipid peroxidation, Mitochondrial citrate synthase activity, mTDNA, Expression levels of peroxisome proliferator-activated receptor gamma-coactivator 1-alpha and transcription factor A	PBMCs
Germain et al. [9]	Fukuda Criteria, IOM 2015	17	15	53.9 (6.2)	51.9 (6.2)	100%	100%	NR	Metabolites	Whole blood
Light et al. [10]	Fukuda criteria	39	22	40–79	40–79	NR	NR	NR	mRNA	Leukocytes
Maes et al. [11]	Fukuda Criteria	58	22	38.5 (13.9)	45.4 (10.1)	86.2%	77.3%	NR	Coenzyme Q10	Plasma
Mandarano et al. [12]	Canadian Criteria	53	45	50.8 (16.2)	50.2 (17.5)	58.5%	57.8%	21.7	Mitochondria	T cells
Missailidis et al. [22]	Canadian Criteria	51	22	26–70	21–58	86%	68%	NR	Mitochondria	Lymphoblasts
Missailidis et al. [23]	Canadian Criteria	51	22	26–70	21–58	86%	68%	NR	Mitochondria	PBMCs Lymphoblasts
Naviaux et al. [13]	Canadian Criteria, Fukuda Criteria, IOM 2015	45	39	F: 52 (2.5) M: 53 (2.8)	F: 48 (2.8) M: 53 (3.5)	51.1%	53.8%	F: 17 (2.3) M: 21 (3.0)	Metabolites	Plasma
Nguyen et al. [14]	Fukuda Criteria	17	19	48.68 (1.06)	46.48 (1.22)	82.4%	68.4%	8.4	TRPM3 surface expression	NK cells, B lymphocytes
Nguyen et al. [15]	Fukuda Criteria, International Consensus Criteria	6	6	50.33 (4.95)	50.00 (5.04)	83.3%	83.3%	NR	Mitochondria	NK cells
Plioplys and Plioplys [16]	Fukuda Criteria	15	15	18–58	19–58	53.3%	53.3%	8 months–20 years	Mitochondria	Percutaneous needle muscle biopsies
Shungu et al. [17]	Fukuda Criteria	15	13	32.7 (8.6)	27.6 (7.4)	80%	53.8%	9.7 (9.1)	Metabolites	Cerebrospinal fluid
Sweetman et al. [18]	Canadian Criteria	10	10	36.4	38.8	60%	60%	12.6	RNA	PBMCs
Tomas et al. [19]	Fukuda Criteria	52	35	42.8 (13.7)	36.6 (12.0)	84.6%	77.1%	NR	Mitochondria	PBMCs
Venter et al. [20]	Fukuda Criteria	UK: 89 moderate, 29 severe RSA: 143 moderate	UK: 64 RSA:98	NR	NR	NR	NR	NR	mtDNA	mtDNA
Yamano et al. [21]	Fukuda Criteria	Training: 47 Validation: 20	Training: 46 Validation: 20	Training: 38.08 (6.57) Validation: 36.15 (8.14)	Training: 38.78 (9.71) Validation: 36.10 (8.35)	Training: 87.2% Validation: 100%	Training: 89.1% Validation: 100%	NR	Metabolites	Plasma

*ATP* adenosine triphosphate, *CoQ10* Coenzyme Q10, *DNA* deoxyribonucleic acid, *Dx* diagnostic criteria, *F* female, *HC* healthy control, *IOM* Institute of Medicine, *M* male, *mtDNA* mitochondrial deoxyribonucleic acid, *ME/CFS/SEID* myalgic encephalomyelitis/chronic fatigue syndrome/systemic exertion intolerance disease, *NK* natural killer, *NR* not recorded, *N* number, *PBMCs* peripheral blood mononuclear cells, *RSA* Republic of South Africa, *RNA* ribonucleic acid, *SD* standard deviation, *TRPM3* transient receptor potential melastatin 3, *UK* United Kingdom

Different sample types were sourced across the studies; four studies used peripheral blood mononuclear cells (PBMCs) [8, 18, 19, 23], three studies used plasma [11, 13, 21], two studies used Natural Killer (NK) cells [14, 15], two studies used lymphoblasts [22, 23], two studies used mtDNA [6, 20], two studies used whole blood [5, 9], one study used cerebrospinal fluid [17], one study used neutrophils [7], one study used urine [5] and one study used percutaneous needle muscle biopsies [16]. From these samples, a variety of different markers were assessed including: metabolites [5, 9, 13, 21], mtDNA [6, 20], Messenger ribonucleic acid (mRNA) [10, 18], ATP, CoQ_10_ [8, 11] or mitochondria directly [12, 15, 16, 19, 22, 23]. Primary outcomes of these studies have been summarized in Table 4.

**Table 4:** Summary of primary outcome results

Author (date)	Technique	Sample	Findings
Armstrong et al. 2015	NMR spectroscopy	Whole blood, urine	Twenty-nine metabolites in blood and thirty metabolites in urine were identified. The absolute concentrations of six blood metabolites were significantly different following NMR analysis. Glucose levels were increased (p = 0.011) in ME/CFS/SEID patients compared with HC. Whereas acetate (p = 0 = 0.04), glutamate (p = 0.029), hypoxanthine (p = 0.001), lactate (p = 0.006) and phenylalanine (p = 0.001) were decreased in ME/CFS/SEID patients compared with HC. Metabolites analysed as a function of total metabolite concentrations reported six metabolites that were significantly different. For this analysis aspartate (p = 0.049) and glucose (p = 0.002) were increased whereas glutamate (p = 0.036), hypoxanthine (p = 0.003), lactate (p = 0.004) and phenylalanine (p = 0.003) were decreased. The absolute concentrations of five urinary metabolites were significantly different in ME/CFS/SEID patients compared with non-ME/CFS/SEID controls using NMR analysis. All urinary absolute concentration metabolites were decreased: acetate (p = 0.003); alanine (p = 0.049); formate (p = 0.002); pyruvate (p = 0.034) and serine (p = 0.034). Eight metabolites were significantly different within relative abundance data. These metabolites were decreased in ME/CFS/SEID patients compared with HC: acetate (p = 0.025); alanine (p = 0.008); formate (p = 0.026); pyruvate (p = 0.001); serine (p = 0.008); valine (p = 0.026). While allantoin (p = 0.011) and creatinine (p = 0.025) were increased
Billing-Ross et al. 2016	Illumina sequencing	DNA	No significant association between mtDNA SNPs and ME/CFS/SEID status compared to HC participants were found. Haplogroups J, U and H (p < 0.01) in addition to eight other SNPs (p < 0.05) were positively correlated with symptoms, symptom clusters or symptom severity in ME/CFS/SEID patients. Overall, heteroplasmy frequency was low in both groups
Booth et al. 2012	ATP profile test	Neutrophils	Using the ATP profile test ME/CFS/SEID patients were found to have measurable mitochondrial disfunction including: ATP availability and oxidative stress efficiency compared to HC participants. No p- value provided
Castro-Marrero et al. 2013	Western blot bioluminescence assay	PBMCs	ME/CFS/SEID patients had significantly lower levels of CoQ_10_ (p < 0.001) and ATP (p < 0.001) and higher levels of lipid peroxidation (p < 0.001) compared to HC participants. Mitochondrial citrate synthase activity and expression levels of mitochondrial DNA content, peroxisome proliferator-activated receptor gamma-coactivator 1-alpha and transcription factor A were not significantly different between the two groups
Germain et al. 2017	Mass spectrometry	Whole blood	74 out of 361 metabolites including energy- related compounds, glucose and oxaloacetate were differentially accumulated in ME/CFS/SEID patients compared to HC participants (p < 0.05). Purines such as ADP and ATP, pyrimidines and many amino acid metabolic pathways were not significantly different between the groups
Light et al. 2013	Real time QPCR	Leukocytes	ME/CFS/SEID patients presented with higher P2X purinoceptor 7 (p = 0.007) and lower Heat Shock Protein Family A (p = 0.032) compared to HC participants. Diazepam binding inhibitor, the gamma-aminobutyric acid A receptor modulator correlated with disease severity for ME/CFS/SEID patients (r = − 0.34, p < 0.05)
Maes et al. 2009	High performance liquid chromatography	Plasma	Compared to HC participants, ME/CFS/SEID patients had significantly lower plasma CoQ10 (p < 0.001). There was a negative correlation between CoQ_10_ levels and total scores on the FF scale (r = − 0.28, p = 0.03), fatigue (r = − 0.86, p < 0.001) and autonomic symptoms (r = − 0.36, p = 0.005)
Mandarano et al. 2019	Seahorse XFe96 Flow cytometry Confocal microscopy	T cells	CD8^+^ T cells belonging to ME/CFS/SEID patients had lower mitochondrial membrane potential (p < 0.01), proton leak (p < 0.05) and ATP production (p < 0.05) compared to HC participants. Glycolysis at rest was lower in CD8^+^ and CD4^+^ cells from ME/CFS/SEID patients (p < 0.05)
Missailidis et al. 2020A	MitoTracker Green FM Seahorse XFe24 (mitochondrial stress test)	Lymphoblasts	ME/CFS/SEID lymphoblasts showed significantly less activation of ATP synthesis by complex V (p = 0.004), mitochondrial membrane potential (p = 0.024), hyperactivated TOR complex 1 stress signalling (p < 0.001) and greater activation of Complex 1 OCR (p = 0.005), maximum OCR (p = 0.002), spare respiratory capacity (p = 0.024), nonmitochondrial OCR (p = 0.002), enzymes of β-oxidation (p < 0.001) and TCA cycles (p = 0.004) as well as proton leak (p = 0.006) compared to HC participants. There was no difference in mitochondrial mass, genome copy number, glycolytic rates and steady state ATP levels between the two groups
Missailidis et al. 2020B	Seahorse XFe24 (mitochondrial stress test) XF glycolysis stress test	PBMCs, Lymphoblasts	Recovered lymphocytes from frozen storage death rate, mitochondrial respiratory function and TORC1 activity can be used as an effective biomarker for ME/CFS/SEID with 90% sensitivity. ME/CFS/SEID patients had a greater lymphocyte death rate compared to HC participants (p < 0.001). Mitochondrial membrane potential, the rate of O_2_ consumption (OCR) by ATP synthesis and the proton leak, the maximum OCR by uncoupled mitochondria, the uncoupled activity of Complex I and the non-mitochondrial OCR values were effectively able to discriminate ME/CFS/SEID patients to HC participants (p < 0.001). The phosphorylation state of TORC1 Kinase Substrate, 4E-BP1can also be used to differentiate between patient and HC groups (p < 0.001)
Naviaux et al. 2016	Hydrophilic interaction liquid chromatography, electrospray ionization, and tandem mass spectrometry	Plasma	Abnormalities in 20 metabolic pathways out of 63 were found in ME/CFS/SEID patients compared to HC participants; this includes, purine (p = 0.044), cholesterol (p = 0.035), pyrroline-5-carboxylate (p = 0.014), riboflavin (p = 0.005) and branch chain amino acid (p = 0.023) metabolism. No p value was recorded
Nguyen et al. 2016	Flow cytometry	NK cells, B lymphocytes	Compared to HC participants, ME/CFS/SEID patients were found to have reduced TRPM3 surface expression on CD19^+^ B cells and CD56^bright^ NK cells (p < 0.05). CD56^bright^ NK cells exposed to 2-APB and thapsigargin had significantly decreased cytoplasmic calcium (p < 0.05)
Nguyen et al. 2019	Seahorse XFp	NK cells	Compared to HC participants, glycolytic reserve in resting NK cells were significantly lower in ME/CFS/SEID patients (p < 0.05). There was no difference in mitochondrial respiration between the two groups
Plioplys and Plioplys 1995	Electron microscopy	Percutaneous needle muscle biopsies	There were no significant mitochondrial abnormalities found between ME/CFS/SEID patients and HC participants including: subsar- colemmal mitochondrial aggregates, intermyofibrillar mitochondrial aggregates, mitochondrial circumference, area, pleomorphism or compartmentalization of the inner mitochondrial membrane
Shungu et al. 2012	Magnetic resonance spectroscopy	Cerebrospinal fluid	No significant differences in high energy phosphate metabolites including, ATP, creatine phosphate (PCr) and inorganic phosphate (Pi), were found between ME/CFS/SEID patients and HC participants
Sweetman et al. 2019	RNA sequencing	PBMCs	Significantly increased gene transcripts important for mitochondrial function, including *PMAIP1, PMPCB* and *JUN,* were found in ME/CFS/SEID patients compared to HC participants (p < 0.001)
Tomas et al. 2017	Seahorse XFp	PBMCs	ME/CFS/SEID patients had significantly lower oxidative phosphorylation parameters including: basal respiration (p ≤ 0.005), ATP production (p ≤ 0.005), proton leak (p ≤ 0.005), maximal respiration (p ≤ 0.05), reserve capacity (p ≤ 0.005), non-mitochondrial respiration (p ≤ 0.005), and coupling efficiency (p ≤ 0.005). Glycolytic activity did not significantly differ between the two groups.
Venter et al. 2019	DNA sequencing	mtDNA	Majority of the severely affected and moderately affected patient groups from South Africa and the United Kingdom did not have a mildly deleterious population variant. Haplogroup distributions and heteroplasmy analysis did not detect any variations of significance between ME/CFS/SEID patients and HC participants across both population groups
Yamano et al. 2016	Agilent CE capillary electrophoresis system	Plasma	Compared to HC participants, ME/CFS/SEID patients exhibited significantly higher intermediate metabolite concentrations including: ornithine/citrulline, pyruvate/isocitrate ratios in the tricarboxylic acid (TCA) and urea cycles (p < 0.001)

*ADP* adenosine diphosphate, *ATP* adenosine triphosphate, *CoQ10* Coenzyme Q10, *DNA* deoxyribonucleic acid, *HC* healthy control, *mtDNA* mitochondrial deoxyribonucleic acid, *ME/CFS/SEID* myalgic encephalomyelitis/chronic fatigue syndrome/systemic exertion intolerance disorder, *NK* natural killer, *NMR* nuclear magnetic resonance, *OCR* oxygen consumption rate, *PBMCs* peripheral blood mononuclear cells; *RNA* ribonucleic acid, *SNPs* single nucleotide polymorphisms, *TRPM3* transient receptor potential melastatin 3, *TCA* tricarboxylic acid, *2-APB* 2-aminoethoxydiphenyl borate

### Literature reporting changes in mitochondrial deoxyribonucleic acid (DNA)/ribonucleic acid (RNA)

One study found no significant single nucleotide polymorphisms (SNPs) between ME/CFS/SEID patients and HC participants. There, however, was a positive correlation between haplotypes J, U and H as well as eight other SNPs and symptoms, symptom clusters or symptom severity in ME/CFS/SEID patients. Heteroplasmy frequency was low [6].

Another study assessed DNA variants in patient groups and HC participants from two distinct locations: South Africa and the United Kingdom. Similarly, most patients with severe or moderate ME/CFS did not have a mildly deleterious population variant [20].

A study on RNA from PBMCs found that there were significantly increased gene transcripts important for mitochondrial function in ME/CFS/SEID patients compared with HC participants including *PMAIP1, PMPCB* and *JUN* [18].

### Literature Reporting structural changes in mitochondria

Structural abnormalities relating to mitochondria in ME/CFS/SEID patients including sub-sarcolemmal mitochondrial aggregates, intermyofibrillar mitochondrial aggregates, mitochondrial circumference, mitochondrial area, mitochondrial pleomorphism, or compartmentalization of the inner mitochondrial membrane were examined in one of the studies included in this review. That study did not identify any significant structural changes in ME/CFS/SEID patient mitochondria compared with HC participants [16].

### Literature reporting changes in mitochondrial respiratory function

Mitochondrial respiratory function was investigated in five of the included studies. There were four studies that identified significant differences in mitochondrial respiratory function between ME/CFS/SEID patients and HC participants. One of these studies found that there was lower mitochondrial membrane potential, lower proton leak (oligomycin, ATP synthase inhibitor, resulting in the depletion of proton motive force), lower ATP production in CD8^+^ T cells from ME/CFS/SEID patients compared with HC participants, also lower glycolysis at rest in both CD8^+^ and CD4^+^ cells from ME/CFS/SEID patients [12, 27].

Another study [22] examining lymphoblasts also showed lower mitochondrial membrane potential in ME/CFS/SEID patients compared to HC participants. There was also lowered activation of ATP synthesis by complex V and hyperactivated target of rapamycin (TOR) complex 1 stress signaling. In contrast to the aforementioned study, in Mandarano *et al*’s study there was greater proton leak, greater complex 1 oxygen consumption rate (OCR), greater maximum OCR (OCR is an indicator of cellular metabolism and fitness) and greater spare respiratory capacity (excess respiratory electron transport chain capacity not being used in basal respiration) [12, 22, 28]. Additionally, in Mandarano et al’s study there was also greater nonmitochondrial OCR (oxygen consuming process) activity, greater number of enzymes of β-oxidation and greater tricarboxylic acid cycle (TCA) activity [12].

The Missailidis et al. study also found no difference in mitochondrial mass, genome copy number, glycolytic rates, or steady state ATP levels between ME/CFS/SEID patients and HC participants [22]. These findings were validated in an associated study that found cells belonging to ME/CFS/SEID patients can be differentiated from HC participant cells based on respiratory function parameters [23].

One study examining PBMCs [19] found all oxidative phosphorylation parameters including basal respiration, ATP production, proton leak, maximal respiration, reserve capacity, non-mitochondrial respiration, and coupling efficiency were significantly lower in ME/CFS/SEID patients compared to HC participants. Similarly to the Missailidis et al. study there was no significant difference in glycolytic activity between both groups [22]. One study contrasted this by finding no difference in mitochondrial respiration in NK cells, however, glycolytic function was significantly lower in ME/CFS/SEID patients compared to HC participants [15].

### Literature Reporting Changes in metabolites

From the 19 studies, 12 investigated metabolic changes in ME/CFS/SEID patients compared to HC participants [5, 7–9, 12, 13, 15, 17, 19, 21–23]. One study investigating metabolic changes in cerebral spinal fluid found no differences in levels of high energy phosphate metabolites including ATP, creatine phosphate, and inorganic phosphate between ME/CFS/SEID patients and HC participants. This study used arterial spin labelling reporting higher ventricular lactate and lower glutathione in the ME/CFS/SEID patient group [17]. As previously mentioned, mitochondrial respiratory tests found that there was lower ATP production in three of the studies [8, 12, 19]. One study, however, reported that there was no difference in steady state ATP levels between groups [22]. An ATP profile study found that there was reduced ATP availability in neutrophils from ME/CFS/SEID patients in comparison to HC participant neutrophils [7]. This finding was supported by another study conducted on PBMCs [8].

A study investigating metabolite concentrations in tricarboxylic acid (TCA) and urea cycles found: ornithine, citrulline, pyruvate and isocitrate ratios were significantly higher in ME/CFS/SEID patients compared to HC participants [21]. Another study found that ME/CFS/SEID patients had higher blood glucose levels and lower blood lactate, urine pyruvate and urine alanine compared to HC participants [5]. In plasma, abnormalities in 20 out of 63 metabolic pathways were found in ME/CFS/SEID patients compared to HC participants: this includes purine, cholesterol, pyrroline-5-carboxylate, riboflavin, and branch chain amino acid [13].

### Literature reporting changes in coenzymes

One study found significantly lower levels of CoQ_10_ in PBMCs from ME/CFS/SEID patients compared to HC participants [8]. Another study supported this, finding FibroFatigue scale scores, fatigue, and autonomic symptoms negatively correlated with CoQ_10_ levels [11].

### Literature reporting changes in mitochondrial signaling pathways

One study investigated cytosol and mitochondrial calcium (Ca^2+^) influx following stimulation with 2-aminoethoxydiphenyl borate (2-APB), and thapsigargin in ME/CFS/SEID patients compared to HC participants. There was significantly lower cytosolic Ca^2+^ ion concentration in CD19^+^ B lymphocytes and CD56^bright^ NK cells in ME/CFS patients compared to HC participants. In ME/CFS/SEID patients, however, there was no significant difference in mitochondrial Ca^2+^ concentration compared to HC participants [14].

### Quality assessment

The JBI CACCCS checklist was used to assess each article for quality and bias (Additional File 2). Items four, five and nine were omitted due to exclusion of interventional studies from this review. Item eight was the most addressed item where 19 (100%) of included studies assessed outcomes in a standard, valid, reliable way. Secondly, 15 (78.9%) of included studies selected appropriate criteria for ME/CFS/SEID and HC participants. From all included studies, 14 (73.7%) appropriately matched patient and HC groups (item one), identified confounding factors (item six), and utilized appropriate statistical tests (item 10). Identified confounding factors were effectively controlled for in 11 (57.9%) of the studies. Checklist item two was least addressed, only 10 studies (52.6%) adequately matched source population.

## Discussion

The aim of this systematic review was to collect and analyze current research on the role of mitochondria in ME/CFS/SEID pathomechanisms. A total of 19 studies met the inclusion criteria and were included in this review. Changes in mitochondrial structure, DNA/RNA, respiratory function, metabolites, and coenzymes have been reported. The results from this systematic review indicate a significant amount of variability across all studies, where in many cases standardized protocols were not apparent or similar outcomes were not assessed, thus making comparisons between studies difficult and ultimately providing little consistency for the analysis of ME/CFS/SEID pathomechanisms.

A systematic review on mitochondrial dysfunction and fatigue was previously published by Filler et al. (2014). That paper used the keyword “fatigue”, however most of the included articles were reporting on ME/CFS/SEID. Since then, numerous papers have been published in the ME/CFS/SEID field. The Filler et al. paper also had less stringent exclusion criteria than this review, included articles that did not have HC participants, had participants under the age of 18 and used criteria beyond the three main established FC, CCC and ICC [29]. The IOMC was not yet established when Filler et al. published in 2014 and has been incorporated to this review. To ensure comparability of the studies examined in this review, stricter exclusion criteria were put in place. This systematic review was the first to assess mitochondrial changes in case control cohorts.

A novel computerized title and abstract screening filter was programmed and implemented in this study by SH. The process of title and abstract screening was made more robust by comparing the automated output against the result from manual title and abstract screening as an additional process verification step, incorporating the advantages of computer processing in a way which did not compromise manual screening. A logically correct computerized title and abstract filter might provide an additional verification layer to reduce human error during title and abstract screening in future studies. Computerized screening may be ideal if the screening process exclusively consists of predefined logical steps without interim decision making, provided the computing architecture is properly designed and operates without error.

In this review, the average ages for ME/CFS/SEID patients and HC participants were 43.7 and 42.4, respectively. Most of the participants were female (77%), this is consistent with literature stating the illness most commonly affects females aged between 35 and 45 [15, 30, 31]. Selected studies matched participants by age and sex [5, 6, 8, 9, 11–13, 15–18, 22, 23]. Matching these characteristics in mitochondrial studies is essential to account for age-related mitochondrial decline and sex-associated differences including uncoupled respiration, citrate synthase activity and ATP levels [32]. Six of the studies included data on race or ethnicity, reporting the majority of participants as Caucasian; this feature is also consistent with literature [15, 30, 31].

The predominant use of the FC across the studies is a significant limitation considering the broad nature of this criteria and considerable overlap with other illnesses [3]. This may explain some inconsistencies found between studies. With the more recent publications, a greater number of papers incorporated the later, more stringent CCC, ICC or IOMC into their recruitment decisions [5, 9, 12, 13, 15, 18, 22, 23]. The inclusion of more stringent criteria allows for a more homogenous subset of patients [3].

Analysis was conducted on samples from a variety of different sources including urine, whole blood, plasma, PBMCs, NK cells, B cells, and T cells. The selection of immune cells resulted from published data demonstrating immune involvement in ME/CFS/SEID pathology [12]. The different cell types make it difficult to establish comparisons between the studies. Cells were from different sources (frozen and fresh), further complicating comparison. For experiments such as measuring mitochondrial respiration, the cellular stress arising from the freezing process significantly impacted cellular bioenergetics including ATP production, maximal respiration, and reserve capacity of the PBMCs in both groups [19].

Only one study included in this review reported on ultrastructural aberrations. These included: extramitochondrial aggregates, mitochondrial circumference, mitochondrial area, and mitochondrial pleomorphism in ME/CFS/SEID patients compared to HC participants [16]. Reports not included in this review described mitochondrial changes in 70% of ME/CFS/SEID patients, involving changes in mitochondrial size as well as cristae branching and cristae fusion resulting in a compartmentalized appearance [33, 34]. Plioplys and Plioplys used similar protocol, differing by criteria used to define ME/CFS/SEID patients, yet reported contradictory findings. This difference may have arisen as a result of inconsistencies in quantification methods [16].

All included DNA studies in this review suggested that ME/CFS/SEID patients do not exhibit disease causing variants [20]. This finding was consistent across both moderately and severely affected ME/CFS patient groups. Additionally, there was no difference found in DNA copy number. A limitation to our search strategy was that some potentially relevant articles were excluded based on not having a HC group. Further analysis of those excluded articles supported these findings (e.g., [35]). These findings suggest that ME/CFS/SEID is not a primary mitochondrial disorder. Altered leukocyte gene expression levels have been identified in fatigue-related pathways [10]. Transcriptome analyses found significantly increased transcription of three genes in ME/CFS/SEID patients: *PMAIP1, PMPCB* and *JUN* [18]. These genes have an important role in mitochondrial function and apoptosis, and may contribute to the neuroinflammatory processes, oxidative stress, increased ventricular lactate, imbalanced metabolites, disrupted circadian rhythm, and impaired respiratory function described across multiple studies [5, 12, 13, 17, 18, 21–23, 35].

Results from respiratory function and glycolysis studies lacked consistency. Interestingly, two papers reported decreased proton leak with decreased mitochondrial efficiency parameters in ME/CFS/SEID patients [12, 19]. An *increase* in proton leak would usually correspond to decreased mitochondrial efficiency, as observed in two other studies by Missailidis et al. [12, 22, 23, 36]. Missailidis et al. showed that respiratory function parameters can be part of an effective method to distinguish between ME/CFS/SEID and HC participant cells, however these findings require further validation in a larger cohort [23]. It is difficult to make reliable conclusions based on the glycolytic activity and respiratory function described in these featured studies, further research is required.

Nguyen et al. found no significant mitochondrial Ca^2+^ concentration changes in the presence of stimulants [14]. That study, however, reported a reduction of cytoplasmic Ca^2+^ concentration in CD19^+^ B lymphocytes and CD56^bright^ NK cells in the presence of stimulants. Mitochondrial processes including respiratory function are Ca^2+^-dependent, cytosolic Ca^2+^ levels influence uptake by mitochondria through Ca^2+^-dependent channels [37, 38]. Disruption of Ca^2+^ channel function, specifically transient receptor potential melastatin 3, has been implicated in ME/CFS/SEID patient NK cell pathology resulting in decreased Ca^2+^ mobilization [39, 40]. As Ca^2+^ is fundamental for many NK cell processes including cytotoxicity, NK cell function is consequently impaired [39, 40]. This impairment may aggravate the production of reactive oxygen species and contribute to the decline of mitochondrial processes, both observed in other studies [41]. Impaired NK cell cytotoxicity is the most consistently described feature in ME/CFS/SEID [42]. Mitochondrial dysfunction may therefore be a consequence, rather than a primary causative factor, in ME/CFS/SEID [44].

Twelve of the studies investigated changes in metabolites including ATP, TCA, and urea cycles [5, 7–9, 12, 13, 15, 17, 19, 21–23]. In this review, discrepancies were observed in ATP levels. The reduction of ATP levels was observed in three studies [8, 12, 19]. Three studies reported no changes in ATP levels [9, 17, 22]. Another study not included in the final review found an increase in externally-derived mitochondria [43]. Missailidis et al. reported that ATP levels were able to be maintained despite inefficiency of Complex V, due to signaling networks being able to homeostatically respond to cellular stresses [17]. Conditions might account for varied results, for example cellular glucose levels [19]. Citrulline, pyruvate, and isocitrate ratios were significantly higher in ME/CFS/SEID patients compared to HC participants. Succinate (which is downstream of the other metabolites) does not significantly differ in level between ME/CFS/SEID patients and HC participants. Disruption of early stages of the TCA cycle is suggested to be present in ME/CFS/SEID patients [21]. Abnormalities in 20 out of 63 metabolic pathways that were tested included: purine, cholesterol, and pyrroline-5-carboxylate. It has been described that these differences make ME/CFS/SEID patients chemically distinguishable from respective HC participants [13]. This is only a small representation of available metabolite studies, a detailed investigation on metabolomic dysregulation in ME/CFS/SEID patients compared to healthy controls has been conducted by Huth et al. [44].

The two studies that investigated CoQ_10_ found a decrease of this compound in ME/CFS/SEID patients compared to HC participants [8, 11]. This reduction could result in an upregulation of nuclear factor kappa-light-chain-enhancer in activated B cells (NFκB) and also induction of oxidative and nitrosative stress pathways [11]. An interventional study has shown potential benefits of CoQ_10_ plus nicotinamide adenine dinucleotide supplementation, finding reduced maximum heart rate and perceptions of fatigue post exercise testing in ME/CFS/SEID patients [45]. Interventional studies, however, were not in the scope of this review.

An article published by Gorman et al. identified overlapping features in classic forms of mitochondrial disease and ME/CFS/SEID, perceived fatigue was a particular feature [46]. Molecular analysis of mitochondrial dysfunction in ME/CFS/SEID has not identified any characteristic mitochondrial gene variants in mitochondrial disease [20]. Smits et al. compared mitochondrial respiratory chain complex activity between ME/CFS/SEID, known mitochondrial disorder and HC participants [47]. Due to the inclusion of inappropriate HC participants, that paper was not included in the final review, it identified distinct differences in ATP production rate and respiratory chain complex activity between ME/CFS/SEID and known mitochondrial disorder participants [47].

An additional study investigating the presence of autoreactive antibodies in ME/CFS/SEID patients has been released. That article, though following all our inclusion criteria, was not included in the final analysis because it was published after we screened for papers. Only one out of 161 ME/CFS/SEID patients were positive for anti-pyruvate dehydrogenase complex antibodies. Anti-mitochondrial antibodies in general were negative in ME/CFS/SEID populations. This research suggests that mitochondrial dysfunction in ME/CFS/SEID patients cannot be explained by the presence of circulating anti-mitochondrial autoantibodies [48].

All included studies, while evidence of mitochondrial pathway disruption is present, are limited by small sample sizes and experiments are not standardized, having low reproducibility. Deriving appropriate conclusions is therefore difficult. Additionally, these studies are case-controlled so only reflect singular points in time. Longitudinal study design may be an important consideration for future studies as differences in mitochondrial function over time may relate to changes in disease activity, symptom presentation, and symptom severity [35].

### Quality assessment

Quality levels varied across studies. All studies assessed outcomes in a standard, valid and reliable way (JBI CACCCS item eight) for ME/CFS/SEID and HC participants using a variety of different methods such as Seahorse, MitoTracker, nuclear magnetic resonance (NMR) spectroscopy, magnetic resonance spectroscopy, DNA/RNA sequencing and electron microscopy. JBI CACCCS item three was the second-most addressed checklist item, selection criteria used to define ME/CFS/SEID and HC cohorts were provided and consistent between both groups. Absent criteria for HC participants was the main reason why some studies did not adhere to this item. JBI CACCCS items one, six and 10 followed. Groups were comparable (other than the presence of disease in ME/CFS/SEID participants and absence of disease in HC participants) through age and sex matching. Identified confounding factors included obesity, sampling time, medication and/or dietary supplements, smoking status, and shift work. Studies that adhered to JBI CACCCS item 10 conducted appropriate statistical analyses including adjusting for multiple comparisons where required and performing normality checks. Mitigation of confounding factors was variously achieved by inclusion of a sampling time range, exclusion criteria (such as shift work, obesity, and smoking status) and ceasing medications and/or dietary supplements for an advised duration prior to study participation. In some cases, studies identified confounding factors but did not adequately control for them. JBI CACCCS item two was least addressed, involving appropriate matching of ME/CFS/SEID patient source population to HC participants included in the study. In all these cases, source population information was not provided. Recommendations for future studies include stricter selection criteria, exclusion based on smoking status, sociodemographic matching, and age and sex matching ME/CFS/SEID patients to HC participants.

## Conclusion

Evidence of potentially disrupted mitochondrial pathways is difficult to establish with certainty due to the use of different sampling methodology. There is consistent genomic research suggesting that ME/CFS/SEID is not a primary mitochondrial disorder, however, mitochondrial decline might occur due to secondary effects of other disrupted pathways. Additionally, findings across the studies were inconsistent. As population samples were small, these results should be interpreted cautiously. The cause of ME/CFS/SEID remains unknown and future studies using the same ME/CFS/SEID diagnostic criteria and analysis methods are required to  determine mitochondrial contribution to ME/CFS/SEID pathomechanisms.

## Supplementary information

**Additional File 1.** Raw search code.

**Additional File 2.** JBI quality assessment table and descriptions.

## Data Availability

All data generated or analysed during this study are included in this published article.

## Abbreviations

ATP: Adenosine Triphosphate
Ca2+: Calcium
CCC: Canadian Consensus Criteria
CFS/ME/SEID: Chronic Fatigue Syndrome/Myalgic Encephalomyelitis/Systemic Exertion Intolerance Disease
CoQ10: Coenzyme Q10
ETC: Electron Transport Chain
HC: Healthy Control
FC: Fukuda Criteria
ICC: International Consensus Criteria
IOMC: Institute of Medicine Criteria
JBI CACCCS: Joanna Briggs Institute Critical Appraisal Checklist for Case Control Studies
MeSH: Medical Subject Headings
mRNA: Messenger Ribonucleic Acid
mtDNA: Mitochondrial Deoxyribonucleic Acid
NK: Natural Killer
OCR: Oxygen Consumption Rate
PBMCs: Peripheral Blood Mononuclear Cells
PRISMA: Preferred Reporting Items for Systematic Reviews and Meta-Analyses
SNPs: Single Nucleotide Polymorphisms
TCA: Tricarboxylic Acid Cycle
2-APB: 2-Aminoethoxydiphenyl Borate
