# Correction to: A systematic review of mitochondrial abnormalities in myalgic encephalomyelitis/chronic fatigue syndrome/systemic exertion intolerance disease

**DOI:** 10.1186/s12967-020-02575-7

**Published:** 2020-10-27

**Authors:** Sean Holden, Rebekah Maksoud, Natalie Eaton-Fitch, Hélène Cabanas, Donald Staines, Sonya Marshall-Gradisnik

**Affiliations:** 1 National Centre for Neuroimmunology and Emerging Diseases (NCNED), Menzies Health Institute Queensland, Griffith University, Gold Coast, Australia; 2 School of Medicine, Griffith University, Gold Coast, Australia; 3 Consortium Health International for Myalgic Encephalomyelitis, Griffith University, Gold Coast, Australia; 4 School of Medical Science, Griffith University, Gold Coast, Australia

## Correction to: J Transl Med (2020) 18:290 10.1186/s12967-020-02452-3

Following publication of the original article [1], the authors identified errors in the Results section, in the Discussion section and in Tables 2 and 4.

## Results section

In the first paragraph of the ‘Participant and study characteristics’ sub-section, the third sentence requires corrections. The updated sentence is given below and the changes have been highlighted in **bold typeface**:

**Seven** of the studies reported race, wherein the largest proportion of participants were Caucasian [6, 8, 12–14, 17, **22**].

In the second paragraph of the ‘Participant and study characteristics’ sub-section, the first sentence requires corrections. The updated sentence is given below and the changes have been highlighted in **bold typeface**:

Different sample types were sourced across the studies; **five** studies used peripheral blood mononuclear cells (PBMCs) [8, 18, 19, 23, **22**], three studies used plasma [11, 13, 21], two studies used Natural Killer (NK) cells [14, 15], two studies used lymphoblasts [22, 23], two studies used mtDNA [6, 20], two studies used whole blood [5, 9], one study used cerebrospinal fluid [17], one study used neutrophils [7], one study used urine [5] and one study used percutaneous needle muscle biopsies [16].

The second paragraph of the ‘Literature reporting changes in mitochondrial respiratory function’ sub-section, requires corrections. The updated paragraph is given below and the changes have been highlighted in **bold typeface**:

Another study [22] examining lymphoblasts also showed lower mitochondrial membrane potential in ME/CFS/SEID patients compared to HC participants. There was also lowered activation of ATP synthesis by complex V and hyperactivated target of rapamycin (TOR) complex 1 stress signaling. **In Missailidis et al’s study [22]** there was greater proton leak, greater complex 1 oxygen consumption rate (OCR), greater maximum OCR (OCR is an indicator of cellular metabolism and fitness) and greater spare respiratory capacity (excess respiratory electron transport chain capacity not being used in basal respiration) [12, 22, 28]. Additionally, in **Missailidis et al’s study** there was also greater nonmitochondrial OCR (oxygen consuming process) activity, greater number of enzymes of β-oxidation and greater tricarboxylic acid cycle (TCA) activity [**22**].

## Discussion section

The third sentence of the eighth paragraph requires a correction. The updated sentence is given below and the change has been highlighted in **bold typeface**:

Additionally, there was no difference found in DNA copy number **[22]**.

The sixth sentence of the eleventh paragraph requires a correction. The updated sentence is given below and the change has been highlighted in **bold typeface**:

Missailidis et al. reported that ATP levels were able to be maintained despite inefficiency of Complex V, due to signaling networks being able to homeostatically respond to cellular stresses [17, **22**].

## Tables

In Table 2, the last column of the ‘Missailidis et al. 2020B’ row requires a correction: the sentence “XF Glycolysis stress test” needs to be removed.

In Table 4, the first sentence in the last column of the ‘Missailidis et al. 2020B’ row requires a correction. The updated sentence is given below and the change has been highlighted in **bold typeface**:

Recovered lymphocytes from frozen storage death rate, mitochondrial respiratory function and TORC1 activity can be used as an effective biomarker **with greater than 90% sensitivity**.
