# Supplementary material for: A systematic review of mitochondrial abnormalities in myalgic encephalomyelitis/chronic fatigue syndrome/systemic exertion intolerance disease
Source: J Transl Med. 2020 Jul 29;18:290. doi: 10.1186/s12967-020-02452-3 (PMC7392668; doi:10.1186/s12967-020-02452-3)
Supplement: Supplementary file 1 — Additional File 1. Raw search code. [file 12967_2020_2452_MOESM1_ESM.docx]

**Raw search code**

Contains FIRST PASS + Pyruvate dehydrogenase + Electron transport chain + ATP + ADP + TCA cycle + Citric acid cycle

Pubmed

"Mitochondria"[Mesh] OR "Mitochondr*"[All Fields] OR "Energy Metabolism"[All Fields] OR “Pyruvate dehydrogenase”[All Fields] OR “Electron transport chain”[All Fields] OR “ATP”[All Fields] OR “ADP”[All Fields] OR “TCA cycle”[All Fields] OR “Citric acid cycle”[All Fields] AND "Fatigue Syndrome, Chronic"[Mesh]

SCOPUS, MEDLINE AND EMBASE

((“Mitochondria”) OR (“Mitochondrion”) OR (“Mitochondrial Contraction”) OR (“Contraction, Mitochondrial”) OR (“Contractions, Mitochondrial”) OR (“Mitochondrial Contractions”) OR ("Mitochondri*") OR (“Energy Metabolism”) OR (“Pyruvate dehydrogenase”) OR (“Electron transport chain”) OR (“ATP”) OR (“ADP”) OR (“TCA cycle”) OR (“Citric acid cycle”)) AND (( "Chronic Fatigue Syndrome") OR ( "Myalgic Encephalomyelitis") OR (“Encephalomyelitis, Myalgic”) OR (“Chronic Fatigue Syndromes”) OR (“Fatigue Syndromes, Chronic”) OR (“Chronic Fatigue-Fibromyalgia Syndrome”) OR (“Chronic Fatigue Fibromyalgia Syndrome”) OR (“Chronic Fatigue Fibromyalgia Syndromes”) OR (“Fatigue-Fibromyalgia Syndrome, Chronic”) OR (“Fatigue-Fibromyalgia Syndromes, Chronic”) OR (“Postviral Fatigue Syndrome”) OR (“Infectious Mononucleosis-Like Syndrome, Chronic”) OR (“Infectious Mononucleosis Like Syndrome, Chronic”) OR (“Royal Free Disease”) OR (“Chronic Fatigue and Immune Dysfunction Syndrome”) OR (“Chronic Fatigue Disorder”) OR (“Chronic Fatigue Disorders”) OR (“Fatigue Disorder, Chronic”) OR (“Fatigue Disorders, Chronic”) OR ( "systemic exertion intolerance" ) OR (“Fatigue Syndrome, Postviral”) OR (“Fatigue Syndromes, Postviral”) OR (“Postviral Fatigue Syndromes”))
