# Supplementary material for: A systematic review of mitochondrial abnormalities in myalgic encephalomyelitis/chronic fatigue syndrome/systemic exertion intolerance disease
Source: J Transl Med. 2020 Jul 29;18:290. doi: 10.1186/s12967-020-02452-3 (PMC7392668; doi:10.1186/s12967-020-02452-3)
Supplement: Supplementary file 2 — Additional File 2. JBI quality assessment table and descriptions. [file 12967_2020_2452_MOESM2_ESM.docx]

JBI quality assessment table and descriptions

|  | 1 | 2 | 3 | 4 | 5 | 6 | 7 | 8 | 9 | 10 |
| --- | --- | --- | --- | --- | --- | --- | --- | --- | --- | --- |
| Armstrong et al 2015 | Yes | No | Yes | N/A | N/A | Yes | No | Yes | N/A | Yes |
| Billing-Ross et al 2016 | Yes | Yes | Yes | N/A | N/A | Yes | Yes | Yes | N/A | Yes |
| Booth et al 2012 | U | No | No | N/A | N/A | No | No | Yes | N/A | No |
| Castro-Marrero et al 2013 | Yes | Yes | Yes | N/A | N/A | Yes | Yes | Yes | N/A | Yes |
| Germain et al 2017 | Yes | No | Yes | N/A | N/A | No | No | Yes | N/A | No |
| Light et al 2013 | Yes | Yes | Yes | N/A | N/A | Yes | Yes | Yes | N/A | Yes |
| Maes et al 2009 | Yes | No | Yes | N/A | N/A | Yes | Yes | Yes | N/A | No |
| Mandarano et al 2019 | Yes | Yes | No | N/A | N/A | Yes | No | Yes | N/A | Yes |
| Missailidis et al 2020A | Yes | Yes | Yes | N/A | N/A | Yes | Yes | Yes | N/A | No |
| Missailidis et al 2020B | Yes | No | Yes | N/A | N/A | Yes | No | Yes | N/A | Yes |
| Naviaux et al 2016 | Yes | Yes | Yes | N/A | N/A | No | No | Yes | N/A | Yes |
| Nguyen et al 2016 | No | Yes | Yes | N/A | N/A | Yes | Yes | Yes | N/A | Yes |
| Nguyen et al 2019 | Yes | Yes | Yes | N/A | N/A | Yes | Yes | Yes | N/A | No |
| Plioplys and Plioplys 1995 | Yes | No | Yes | N/A | N/A | Yes | Yes | Yes | N/A | Yes |
| Shungu et al 2012 | Yes | U | Yes | N/A | N/A | Yes | Yes | Yes | N/A | Yes |
| Sweetman et al 2019 | Yes | Yes | Yes | N/A | N/A | No | No | Yes | N/A | Yes |
| Tomas et al 2017 | No | No | No | N/A | N/A | Yes | Yes | Yes | N/A | Yes |
| Venter et al 2019 | No | Yes | No | N/A | N/A | No | No | Yes | N/A | Yes |
| Yamano et al 2016 | U | No | Yes | N/A | N/A | Yes | Yes | Yes | N/A | Yes |

Supplementary Table: The Joanna Briggs Institute Checklist for Case Control Studies. Items answered as not applicable were removed from the final percentage. Abbreviations: JBI, Joanna Briggs Institute; Y, Yes; N, No; N/A, not applicable; U, unclear.

JBI Checklist items:
1. Were the groups comparable other than the presence of disease in cases or the absence of disease in controls?
2. Were cases and controls matched appropriately?
3. Were the same criteria used for identification of cases and controls?
4. Was exposure measured in a standard, valid and reliable way?
5. Was exposure measured in the same way for cases and controls?
6. Were confounding factors identified?
7. Were strategies to deal with confounding factors stated?
8. Were outcomes assessed in a standard, valid and reliable way for cases and controls?
9. Was the exposure period of interest long enough to be meaningful?
10. Was appropriate statistical analysis used?

**Armstrong et al, 2015**

1. Age and sex- matched.
2. Source population information not provided.
3. ME/CFS patients defined according to the Canadian Consensus Criteria. HCs were not suffering from ME/CFS or any other illness. No subjects were obese or nicotine dependent.
4. No exposure. Study investigated metabolic profiles of ME/CFS patients compared to HCs using NMR spectroscopy.
5. As above.
6. Confounding factors include age, sex, medication, oral supplements, obesity or nicotine dependency.
7. Age and sex were catered for by only including a certain age range and females. Medication and oral supplements were noted but how they were addressed was not described. Obese or nicotine dependent participants were excluded from the study.
8. Yes, outcomes were measured using NMR spectroscopy.
9. No exposure.
10. T- tests with Benhamini- Hochberg adjustments were conducted to detect significant metabolites. These significant metabolites were compared with their respective samples using Pearson correlation.

**Billing-Ross et al 2016**

1. Age, ethnicity and sex- matched.
2. Location radius was in the United States (Miami, Salt Lake City, Boston, New York City, and Sierra). Participants were ethnicity matched.
3. Patients were defined by either the 1994 Fukuda criteria or the Canadian Consensus Criteria. Healthy controls were recruited if mentally and physically healthy.
4. No exposure. Study investigated mitochondrial DNA variants in ME/CFS patients compared to HCs.
5. As above.
6. Time of sampling.
7. Sampling time was matched within 12 weeks.
8. Yes, illumine sequencing of PCR- amplified mtDNA was conducted.
9. No exposure.
10. Multiple test corrections were considered using the Benjamini- Hochberg approach to calculate q values.

**Booth et al, 2012**

1. Unclear whether age and sex matched.
2. Source population information not provided.
3. ME/CFS patients were diagnosed according to the Fukuda criteria. HCs not defined.
4. No exposure. Study investigated ATP profiles in ME/CFS patients compared to HCs.
5. As above.
6. Confounding factors not described.
7. As above.
8. Yes, a clinically used ATP profile test was used.
9. No exposure.
10. No statistical methodology was provided.

**Castro-Marrero et al, 2013**

1. Age and sex-matched
2. All participants were Caucasian
3. ME/CFS patients were diagnosed according to the Fukuda criteria. Healthy controls had no symptoms of fibromyalgia or ME/CFS. No associated comorbid phenomena were free from medication at least one month prior to experimentation.
4. No exposure. Study investigated mitochondrial function in ME/CFS patients compared to HCs.
5. As above.
6. Medications
7. All patients and control subjects ceased medication one month prior to experimentation
8. Yes, standardised assays, MtDNA and Western blot were used.
9. No exposure.
10. Association between different groups were analysed using the chi square test.

**Germain et al, 2017**

1. Age, sex and BMI matched.
2. No source population information provided.
3. ME/ CFS patients met Fukuda and IOM 2015 criteria.
4. No exposure. Study investigated metabolic profiles in ME/CFS patients compared to HCs.
5. No exposure.
6. No confounding variables identified.
7. As above.
8. Yes, the study utilises mass spectrometry.
9. No exposure.
10. While the study included information on what software package was used to complete statistical analysis no details on the statistical tests used were disclosed.

**Light et al, 2013**

1. Age and Gender were adjusted for (used as covariates)
2. Participants were from Utah – 97% white and 3% Hispanic
3. ME/CFS patients met the Fukuda criteria. HCs could only report one of the following symptoms that reduce quality of life: easy fatigability, reduced muscle strength, sleep or mood disturbances, need for daytime rest, increased memory or concentration problems and decreased ability to exercise or be active.
4. No exposure: study investigated leukocyte gene expression profiles associated with fatigue in ME/CFS patients compared to HCs using Q RT PCR.
5. No exposure.
6. Age, sex and BMI were identified as potential confounding factors.
7. These factors were statistically adjusted for as covariates.
8. Yes Q RT PCR was used.
9. No exposure.
10. 3- group ANOVA – age and gender were listed as covariates. 46 set of genes were tested for and to adjust for multiple comparisons the alpha level was set at P = 0.025 for the initial ANCOVA comparing the 3 groups and for the stepwise regression models. P = 0.05 for all other analyses.

**Maes et al, 2009**

1. There were no significant differences in age or gender distribution in ME/CFS patients compared to HCs.
2. ME/CFS patients were outpatients admitted to the Mae’s clinic in Belgium, but no information was provided regarding where the HCs were sourced from.
3. ME/CFS patients were diagnosed according to the CDC Fukuda criteria. Patients and control groups also followed the same exclusionary criteria for example: subjects with life-time diagnoses of psychiatric DSM IV-R disorders e.g. depression. Any other medical diagnosis sch as inflammatory bowel disorders, diabetes or hypertension.
4. No exposure. Study investigated levels of Coenzyme Q10 in ME/CFS patients compared to HCs.
5. As above.
6. Abnormal blood tests (ALT, ALP, blood urea nitrogen, calcium, creatinine, electrolytes, TSH and positive antibodies for EBV or CMV. Medications such as beta-blockers or statins or those who take dietary supplements with CoQ10, antipsychotics, anticonvulsants or mood stabilizers. Any participant that had an acute infection at least 2 months prior to the study.
7. All participants with the following were excluded.
8. Yes, Plasma CoQ10 was measured using a HPLC method formulated by Chromsystems Diagnostics.
9. No exposure.
10. Differences in group men was assessed using ANOVA or ANCOVA. Normality or controlling for multiple comparisons was not addressed.

**Mandarano et al, 2019**

1. Age, sex matched.
2. Patients and HCs were ethnicity and race matched.
3. Canadian Consensus Criteria was used to diagnose ME/CFS patients. HC criteria not described.
4. No exposure. This study investigated glycolysis, mitochondrial respiration in resting and activated T cells belonging to ME/CFS patients and HCs as well as markers related to cellular metabolism and plasma cytokines.
5. As above.
6. Medications were identified as a potential confounding factor.
7. Was not controlled.
8. Yes, commercialised Agilent Seahorse XFe96 and a MitoStressTest was used in combination with flow cytometry and confocal microscopy.
9. No exposure.
10. Spearman’s correlation test was used to assess whether there was a correlation with T cell metabolism and plasma cytokine or survey data, a FDR correction for multiple testing was used.

**Missailidis et al, 2020A**

1. Age and sex- matched
2. All participants were of European descent.
3. ME/CFS patients met the Canadian Consensus Criteria. HCs had no family history of ME/CFS or similar myalgias.
4. No exposure. Mitochondria function was assessed using Seahorse extracellular flux analysis in ME/CFS patients compared to HCs.
5. No exposure.
6. Use of lymphocytes were identified as a potential confounding variable as ME/CFS lymphocytes death rate was significantly higher and decreased mitochondrial function may be shown due to a higher fraction of dead cells in the ME/CFS population.
7. To counteract this result – lymphoblastoid cell lines were used.
8. Yes, commercial mitochondrial stress test kits and extracellular flux analyser, Seahorse XFp was used.
9. No exposure.
10. Two sample *t-*tests and binominal t tests were used where appropriate. No adjustments for multiple comparisons were made.

**Missailidis et al, 2020B**

1. Age and sex- matched
2. No source population information provided.
3. ME/CFS patients met the Canadian Consensus Criteria. HCs had no family history of ME/CFS or similar myalgias.
4. No exposure. This study identifies three different potential biomarkers: lymphocyte death rate, mitochondrial respiratory function and TORC1 activity
5. No exposure.
6. Freezing ME/CFS patients was identified as a potential confounding variable resulting in increased cell death in ME/CFS cell cultures.
7. Confounding factor identified but this was not controlled for.
8. Yes, commercial mitochondrial stress test kits and extracellular flux analyser, Seahorse XFp was used.
9. No exposure.
10. Usefulness of biomarker was statistically assessed using AUC with 95% confidence limits.

**Naviaux** **et al, 2016**

1. Age, demographics and sex- matched.
2. Patients and HCs were matched based on demographics.
3. Patients met 2015 IOM, Canadian and Fukuda diagnostic criteria for ME/CFS. HCs did not have ME/CFS.
4. No exposure. Study investigated metabolic features of ME/CFS compared to HCs using hydrophilic interaction liquid chromatography.
5. As above.
6. No confounding factors were identified.
7. As above.
8. Yes, metabolic features were assessed using hydrophilic interaction liquid chromatography.
9. No exposure.
10. Univariate ANOVA with pairwise comparisons post hoc to account for multiple comparisons were made.

**Nguyen et al, 2016**

1. This study was age but not sex matched.
2. All participants were Caucasian and were residents of Australia.
3. Patients met Fukuda criteria. HCs did not have ME/CFS criteria. Exclusion criteria for all participants included those who were smokers, pregnant or breastfeeding.
4. No exposure. Study investigated TRPM3 surface expression and cytoplasmic and mitochondrial calcium influx in CD19+ B cells, CD56^bright^ and CD56^dim^ cell populations in ME/CFS patients compared to HCs.
5. As above.
6. Pathology testing parameters
7. There were no significant differences.
8. Yes, flow cytometry was used.
9. No exposure.
10. MANOVA was used. The levene test was employed to analyse homogeneity of variance between ME/CFS patients and HCs. Post hoc test was used to adjust for multiple comparisons.

**Nguyen et al, 2019**

1. Age and sex – matched
2. Source population from South-East Queensland region of Australia.
3. Participants met both Fukuda and International consensus criteria. Both ME/CFS patients and HCs had no exclusionary medical conditions including autoimmune disease, cardiovascular disease, primary psychiatric disorders, pregnancy and smokers.
4. No exposure. Study investigated glycolytic reserve in isolated natural killer cells from ME/CFS patients and HCs.
5. As above.
6. Smoking, medications such as immune regulators and hormone therapy.
7. Smokers and those who took immune regulators and hormone therapy were excluded.
8. Yes, commercial mitochondrial stress test kits and extracellular flux analyser, Seahorse XFp was used.
9. No exposure.
10. Shapiro- Wilk analysis and Leven’s test were used to determine normality and homogeneity. One-way ANOVA was used to determine demographic characteristics, blood parameters and difference in mitochondrial respiration or glycolytic function in NK cells between HCs and ME/CFS patients. Adjustment for multiple comparisons was not made.

**Plioplys and Plioplys et al, 1995**

1. Age and sex matched
2. Source population information not provided.
3. Patients met CDC Fukuda criteria. The controls did not have any significant medical or psychiatric illnesses.
4. No exposure. This study investigated ultrastructural mitochondrial abnormalities in ME/CFS patient compared to HCs using electron microscope.
5. As above.
6. Day-to-day technical differences from biopsy.
7. All muscle biopsies were done on the same day with ME/CFS patient and their respective HC.
8. Yes, the muscle biopsies were assessed using electron microscope.
9. No exposure.
10. For analysis student’s two tail t test, discriminant function analysis, ANOVA and Pearson’s rank correlation coefficients were used.

**Shungu et al. 2012**

1. Age and sex matched to ME/CFS patients.
2. HC’s were recruited through local media advertisement. CFS patients were recruited via clinician referrals and media advertisements. Unclear of location radius of CFS patients.
3. Patients were diagnosed according to the modified US Centers for Disease Control and Prevention (CDC). HCs did not meet the criteria for ME/CFS or any psychiatric disorder.
4. No exposure. Study investigates the relationship between cortical glutathione and clinical symptoms in ME/CFS patients compared to HCs.
5. As above
6. Lactate CH3 group has nearly the same MR frequency as the ethanol 1.3ppm
7. The participants stopped drinking alcohol at least 48 hours prior to the study commencing.
8. Yes, MRSI and structural MRI were used.
9. No exposure.
10. Normality of data was assessed using the Shapiro- Wilks test. Bonferroni correction was applied for multiple comparisons. For all normally distributed secondary outcome measures three-group comparisons were performed using ANOVA followed by post-hoc comparisons with Tukey’s honestly significant difference test. two-tailed, with the level of significance set at p<0.05.

**Sweetman et al, 2019**

1. Age and sex- matched
2. ME/CFS patients and HCs came from a New Zealand cohort.
3. ME/CFS patients were defined according to the Canadian Consensus Criteria. HCs had no history of significant illness, injury or fatigue related disorders.
4. No exposure. Study investigated transcriptome of circulating immune cells in ME/CFS patients compared to HCs.
5. No exposure.
6. No confounding factors identified.
7. As above.
8. Yes, RNA-seq was conducted according to established protocol.
9. No exposure.

**Tomas et al, 2017**

1. Participants were not age-matched or sex-matched.
2. No source information provided.
3. Patients fulfilled Fukuda criteria. Criteria for healthy controls was not provided.
4. No exposure. Study investigated cellular bioenergetics in patients with ME/CFS compared to HCs.
5. As above.
6. Age was a confounding factor for mitochondrial function. Sex had no effect on maximal respiration so therefore wasn’t a confounding factor. Freezing and thawing of cells contributes to changes in oxidative phosphorylation.
7. Age was omitted as a confounding factor through use of Pearson’s correlation. Fresh samples were also included in addition to frozen to understand the contribution freezing cells has on mitochondrial function.
8. Yes, a commercial reputable mitochondrial stress test kit was used.
9. No exposure.
10. Two-way ANOVA with least significant difference (LSD) test and post-hoc Bonferrroni correction for multiple comparisons were used when required. Anderson-Darling test for normality was used to ensure normal distribution prior to assess whether the dataset had equal variances. Where required, Pearsons correlations were also used.

**Venter et al, 2019.**

1. Not age and sex-matched used regionally matched controls from a previous non-associated study.
2. Study included regionally matched controls
3. ME/CFS patients were defined according to the Fukuda definition. No criteria provided for HCs.
4. No exposure. Study investigated MtDNA variants in ME/CFS patients compared to HCs.
5. As above.
6. No confounding factors were described.
7. As above.
8. Yes, sequencing was carried out using a fluidigm technology.
9. No exposure.
10. Haplogroup distribution and statistical significance was determined using a Monte Carlo approach. Monte Carlo controls for false discovery rate or familywise error rate. Fishers exact tests were then used to compare patients and controls in each cohort that have mtDNA variants.

**Yamano et al, 2016**

1. Unclear if age or sex- matched.
2. No source population information provided.
3. ME/ CFS patients were defined according to Fukuda criteria. All participants psychiatric disorders or chronic diseases that are sometimes accompanied by fatigue e.g cancer or diabetes were excluded. HCs that were shift workers were also excluded.
4. No exposure. Study investigated metabolomic profiles in ME/CFS patients compared to HCs.
5. As above.
6. Shift workers and subjects taking medication that affect autonomic nerve function or the central nervous system.
7. Shift workers and subjects taking medication that affect autonomic nerve function or the central nervous system were excluded from the study.
8. Yes, an agilent CE capillary electrophoresis system was used.
9. No exposure.
10. Mann-Whitney-U test was used to assess statistical significance. Demographic data were compared using Student’s *t-*test and categorical data were compared using Fisher’s exact test.
